# Calcium/Calmodulin–Dependent Protein Kinase II in Cerebrovascular Diseases

**DOI:** 10.1007/s12975-021-00901-9

**Published:** 2021-03-13

**Authors:** Xuejing Zhang, Jaclyn Connelly, Edwin S. Levitan, Dandan Sun, Jane Q. Wang

**Affiliations:** 1 Department of Pharmacology and Chemical Biology, University of Pittsburgh School of Medicine, E1354 BST, Pittsburgh, PA USA; 2 Department of Neurology, Pittsburgh Institute For Neurodegenerative Diseases, University of Pittsburgh, 7016 Biomedical Science Tower-3, 3501 Fifth Ave., Pittsburgh, PA 15260 USA

**Keywords:** Cerebrovascular disease, CaMKII, Stroke, Vascular dementia, Neuronal cell death, Neuroinflammation, Endothelial barrier dysfunction

## Abstract

Cerebrovascular disease is the most common life-threatening and debilitating condition that often leads to stroke. The multifunctional calcium/calmodulin-dependent protein kinase II (CaMKII) is a key Ca^2+^ sensor and an important signaling protein in a variety of biological systems within the brain, heart, and vasculature. In the brain, past stroke-related studies have been mainly focused on the role of CaMKII in ischemic stroke in neurons and established CaMKII as a major mediator of neuronal cell death induced by glutamate excitotoxicity and oxidative stress following ischemic stroke. However, with growing understanding of the importance of neurovascular interactions in cerebrovascular diseases, there are clearly gaps in our understanding of how CaMKII functions in the complex neurovascular biological processes and its contributions to cerebrovascular diseases. Additionally, emerging evidence demonstrates novel regulatory mechanisms of CaMKII and potential roles of the less-studied CaMKII isoforms in the ischemic brain, which has sparked renewed interests in this dynamic kinase family. This review discusses past findings and emerging evidence on CaMKII in several major cerebrovascular dysfunctions including ischemic stroke, hemorrhagic stroke, and vascular dementia, focusing on the unique roles played by CaMKII in the underlying biological processes of neuronal cell death, neuroinflammation, and endothelial barrier dysfunction triggered by stroke. We also highlight exciting new findings, promising therapeutic agents, and future perspectives for CaMKII in cerebrovascular systems.

## Introduction

Cerebrovascular disease affects blood vessels and cerebral circulation of the brain and is a common cause of death and disability worldwide. Stoke, either ischemic or hemorrhagic, is a major form of cerebrovascular disease. In the USA, stroke reduces mobility in more than half of stroke survivors age 65 and over, and approximately 3% of males and 2% of females reported that they were disabled because of stroke [1]. Subarachnoid hemorrhage (SAH), a less common type of hemorrhagic stroke, accounts for 5% of stroke cases in the USA [2]. Severe reduction of cerebral blood flow also leads to a complex neurodegenerative disorder known as vascular dementia which associates with memory and cognitive impairment. Stroke as a major form of cerebrovascular disease has been intensely studied with the primary focus on neuronal injury, the main functional deficit of the disease. However, there is growing appreciation in the functional interactions within the multicellular neurovascular unit and their crucial roles in the onset, progression, and recovery after ischemic stroke [3, 4].

CaMKII is a family of calcium (Ca^2+^) and calmodulin (CaM)–activated multifunctional serine/threonine kinases [5]. It was first discovered in the brain and subsequently shown to play a critical role in brain function, particularly in memory and learning [6]. In ischemic stroke, CaMKII was found to be activated in the early phase of ischemic insult and mediates glutamate excitotoxicity–induced cell death in neurons. However, downstream events leading to cell death remain obscure. Additionally, past studies mainly focus on CaMKII holoenzyme and CaMKIIα (the most abundant CaMKII isoform in the brain) in neurons, but there is sparse information on CaMKII in other cell types within the neurovascular unit (such as endothelial cells, pericytes, smooth muscle cells, astrocytes, microglia, and extracellular matrix) and the functions of other CaMKII isoforms in ischemic stroke are largely unknown. In this review, by revisiting several key biological processes triggered by ischemic or hemorrhagic brain injury, we seek to discuss the diverse functions and signaling mechanisms of CaMKII in cerebrovascular disease in the context of the multicellular neurovascular environment.

## Structure and Regulation of CaMKII

### Basic Enzyme Structure and Classification

**Fig. 1:**
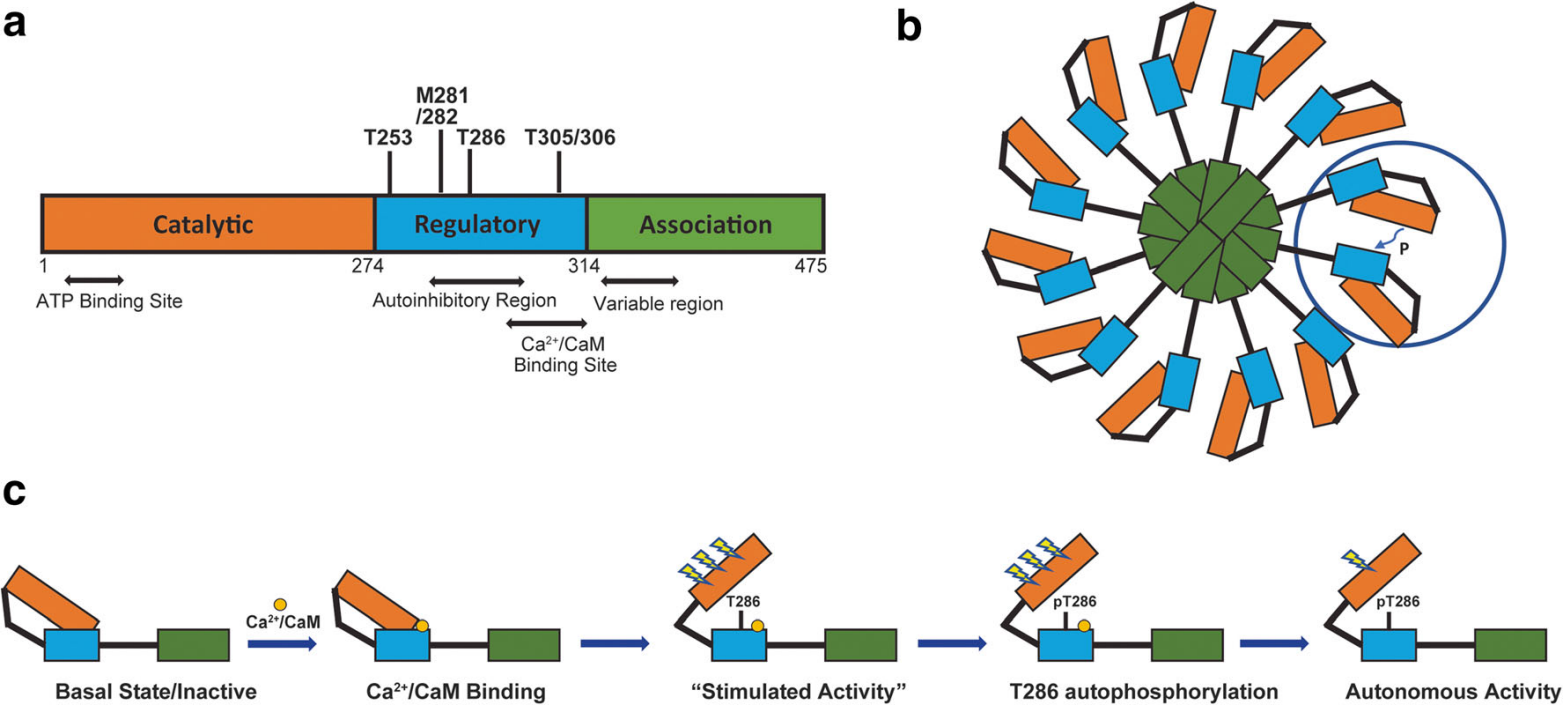
Diagram of CaMKII structure and mechanisms of activation. **a** The sequence of CaMKII includes a N-terminal catalytic domain that contains the ATP binding and substrate phosphorylation sites, a regulatory domain that contains the autoinhibitory region, the Ca^2+^/CaM binding site, and multiple post-translational modification sites including phosphorylation sites T253, T286, and T305/306 and the M281/282 oxidation sites, and a C-terminal association domain that mediates multimeric interactions and a variable region that can undergo alternative splicing to generate a variety of CaMKII subtypes. **b** Schematic depiction of CaMKII holoenzyme with 12 subunits. **c** Activation mechanism of a CaMKII monomer by the binding of Ca^2+^/CaM and autophosphorylation at T286

CaMKII is a family of serine/threonine kinases activated by Ca^2+^ and CaM. Four isoforms (α, β, γ, and δ) have been identified. Each CaMKII holoenzyme typically comprises 12 or 14 subunits of the same or different isoforms [7–11]. The homo- or heteromeric structures confer unique properties to CaMKII based on which isoforms and/or subtypes are present [8]. As shown in Fig. 1, the conserved basic structure of CaMKII includes a N-terminal catalytic domain that contains the ATP binding site, a regulatory domain that encompasses an autoinhibitory region, multiple conserved phosphorylation sites, and a Ca^2+^/CaM binding site required for activation, and a C-terminal association domain that includes a variable region and an association motif that is essential for oligomerization [7–10, 12]. CaMKII is a key regulator of neuronal function and has been studied extensively in areas of learning, synaptic plasticity, and memory [6, 7].

The four isoforms of CaMKII all have conserved domain structures but are derived from different genes [7]. It is a common occurrence for tissues to express more than one isoform of CaMKII [10]. The variable region provides the main structural distinctions between isoforms. Further diversification occurs as a result of alternative splicing in the variable region to produce around 40 different subtypes for the four isoforms [12–14].

All four CaMKII isoforms reside in the brain, though CaMKIIα and CaMKIIβ are known to be more prevalent there, while CaMKIIδ is more abundant in the cardiovascular system and CaMKIIγ is ubiquitously expressed [8–10]. In particular, CaMKIIα is most prevalent in neurons and accounts for more than 1% of protein in some areas of the brain such as the hippocampus and postnatal forebrain neurons [7, 9].

### Mechanisms of Activation

In the absence of stimuli, each subunit of CaMKII is kept in a basal/inactive state by the interactions between its catalytic domain and the autoinhibitory region within the regulatory domain [9]. The alpha helical structure of the autoinhibitory region contains two sequences that overlap with each other, an auto-inhibition sequence and a CaM binding sequence [7, 9]. The conserved threonine 286 (T286) (T287 for CaMKIIβ, δ, γ) phosphorylation site resides in the autoinhibition sequence, and two other conserved phosphorylation sites, T305/306, reside in the Ca^2+^/CaM binding sequence in the regulatory domain (Fig. 1a) [7–9, 12]. The T286 amino acid binds to a conserved site in the catalytic domain to maintain the inactive conformation.

The activation of the CaMKII is triggered by an increase in intracellular Ca^2+^ levels and the binding of Ca^2+^ to CaM to form the Ca^2+^/CaM complex. Ca^2+^/CaM then binds to the Ca^2+^/CaM binding site in the regulatory domain to induce a conformational change that allows the catalytic domain to be separated from the autoinhibitory region and produce what is called “stimulated activity” [7, 8]. This will expose the substrate binding site in the catalytic domain to complete the activation [8]. Individual subunits of CaMKII are activated separately in the holoenzyme [7]. The conformational change induced by Ca^2+^/CaM binding also exposes the conserved T286 phosphorylation site in the autoinhibitory region, allowing it to be phosphorylated by an adjacent, activated subunit. The phosphorylation of T286 prevents the re-binding of the autoinhibitory region to the catalytic domain after Ca^2+^/CaM dissociation. This leads to “constitutive/autonomous activity” [6]. Autophosphorylation of T286 also creates the circumstance of CaM trapping, meaning that the Ca^2+^/CaM complex has reduced dissociation*,* contributing to more activity of the kinase [8].

CaMKII activity is regulated by both phosphorylation and dephosphorylation events. As described above, autophosphorylation of T286 plays a critical role in regulating CaMKII activity. It also regulates the binding capacity of CaMKII, particularly at the post-synaptic density (PSD) [15]. In addition to T286, CaMKII activity can also be regulated by additional autophosphorylations at T305/306 (T306/307 for CaMKII β, γ, and δ) and T253 with CaMKIIα (T254 for CaMKIIβ) [9, 16, 17]. As shown in Fig. 1a, T305/306 is located within the Ca^2+^/CaM binding site and can only become phosphorylated when the Ca^2+^/CaM dissociates and autonomous activation is in effect. When this phosphorylation occurs, the Ca^2+^/CaM complex can no longer bind to cause further stimulation of the kinase, leaving the kinase partially active as a result of autonomous activity due to phosphorylated T286 [16]. The phosphorylation of T253 has no direct effect on the kinase activity or Ca^2+^/CaM binding of purified CaMKII *in vitro*, but affects the targeting of CaMKII via interactions with other binding proteins, therefore has functional effects on cell physiology [9, 16–19]. Dephosphorylation returns the enzyme to an inactive state and is catalyzed by protein phosphatase types 1 and 2A [6, 20–23].

### Major Regulatory Mechanisms of CaMKII

#### RNA Splicing

Alternative splicing of the variable region in CaMKII isoforms gives rise to the different subtypes of the kinase [24]. The splicing confers unique properties to certain subtypes of CaMKII isoforms and allows them to regulate distinct cellular processes. The exact numbers of subtypes for each CaMKII isoform and their functions are still an area of active investigation. Based on current knowledge, RNA splicing produces at least three splice variants for CaMKIIα (α, αB, and αKAP). The α and αB subtypes are prevalent in the brain, while the catalytic domain deficient αKAP subtype is predominantly expressed in the skeletal muscle. There are at least six splice variants for CaMKIIβ including β, β’, βe, and βe’, which are mainly distributed in the brain. CaMKIIδ has at least eleven splice variants (CaMKIIδ_1–11_, including δ_1/A_, δ_3/B_, δ_2/C_, and δ_9_), and CaMKIIγ has at least eight splice variants (γ_A_–γ_H_) [24, 25]. CaMKIIγ and CaMKIIδ subtypes can be found in various locations such as the brain, skeletal muscle, heart, and lung [26, 27]. Within the heart, different δ subtypes have been shown to function differently, e.g., the nuclear CaMKIIδ_B_ controls the expression of the atrial natriuretic factor, and the cytoplasmic CaMKIIδ_C_ controls Ca^2+^ release from the sarcoplasmic reticulum by phosphorylating its cardiac ryanodine receptors [28, 29]. Certain CaMKIIγ subtypes contain a nuclear localization sequence (NLS) signal, which plays a role in shuttling Ca^2+^/CaM to the nucleus to enable the activation of the cAMP-response element binding protein in the brain [30]. A detailed review about CaMKII alternative splicing is provided by Sloutsky and Stratton [14].

#### Subcellular Localization

The localization of CaMKII in cells plays a crucial role in regulating CaMKII function. It has been well documented that certain subtypes of the CaMKII isoforms are capable of nuclear localization (αB, δ_B_, γ_A’_) [30–32]. These CaMKII subtypes share a common core NLS sequence, KKRK, in the variable region to mediate nuclear translocation. This nuclear localization event is also negatively regulated by the phosphorylation of a serine residue immediately adjacent to NLS [25, 30, 33]. Another major regulator of CaMKII localization in cells is the influx of Ca^2+^. In neurons, Ca^2+^ influx triggered by glutamate binding to N-methyl-D-aspartate (NMDA) receptors causes translocation of CaMKII to post-synaptic sites and extra-synaptic clusters for regulation of synaptic plasticity [7]. CaMKII can also be mobilized to other subcellular locations such as the cytoskeleton due to CaMKIIβ binding to F-actin [34]. Upon Ca^2+^ influx, CaMKIIβ will dissociate from the F-actin and translocate to the PSD. It is also common to see a heteromeric structure of α/β-CaMKII bound to F-actin through its interaction with the β subunits [35].

#### Oxidative Stress

Oxidative stress, a condition caused by the overproduction of reactive oxygen species (ROS), can induce oxidative damages on lipids and proteins [36]. ROS has been shown to induce oxidative modifications on a conserved methionine pair M281/282 (C281/M282 in CaMKIIα) in the autoinhibitory region of CaMKII (Fig. 1a) to produce an oxidized form of CaMKII [37]. This oxidation event occurs after Ca^2+^ activation of the kinase when the autoinhibitory region is removed from the catalytic domain. The oxidized methionine pair will keep the autoinhibitory region from re-binding with the catalytic domain after Ca^2+^/CaM dissociation, which creates an autonomously active state similar to that seen with T286 autophosphorylation [38]. Oxidized CaMKII production is counterbalanced by the activity of methionine sulfoxide reductase A which reduces methionine sulfoxide, product of the first oxidation step. Valine mutation for both methionine oxidation sites of CaMKII reduces atrial fibrillation, similar to methionine sulfoxide reductase overexpression, indicating a pathological role for oxidized CaMKII in cardiac disease [39].

#### Protein-Protein Interaction

Protein-protein interactions facilitate the recruitment of CaMKII to different subcellular locations where selective sets of CaMKII substrates are available. Cellular membranes are a major site of protein interactions for CaMKII. In the neurons, one of the most important binding partners of CaMKII is the NMDA receptor subunit GluN2B (NR2B) located at post-synaptic sites, which binds and recruits CaMKII to the synapse after its activation by glutamate stimuli [7]. This interaction is mapped to the T286 site in CaMKII where the accessibility of this site is critical for the binding [40, 41]. A variety of other protein binding partners of CaMKII have also been identified such as the connexin36 which binds and recruits CaMKII to gap junctions [42] and the α subunit of L-type Ca^2+^ channels which interacts with CaMKII at the plasma membrane [43]. Besides membrane association, CaMKII also binds to a F-actin binding protein α-actinin to localize to the cytoskeleton [44].

#### Non-coding RNAs

Non-coding RNAs (ncRNA) are functional RNAs but do not encode proteins. ncRNAs play important roles in transcriptional and post-transcriptional regulation of gene expression. Two major groups of ncRNAs, microRNAs which are short RNAs less than 200 nucleotides in length and long non-coding RNAs (lncRNAs) which are longer than 200 nucleotides, have both been implicated in regulating CaMKII gene expression [45]. MicroRNA-145 downregulates the expression of CaMKIIδ in cardiomyocytes and reduces Ca^2+^ overload [46]. Our group identified two novel *CAMK2D*-associated lncRNAs, *CAMK2D*-associated transcript 1 (*C2dat1*) and *CAMK2D*-associated transcript 2 (*C2dat2*), that are induced by ischemic insults in the neurons and regulate CaMKIIδ expression in response to ischemia/reperfusion (I/R) [47, 48]. The mechanisms through which these ncRNAs regulate CaMKII gene expression remain to be fully elucidated.

## CaMKII and Cerebrovascular Diseases

**Fig. 2:**
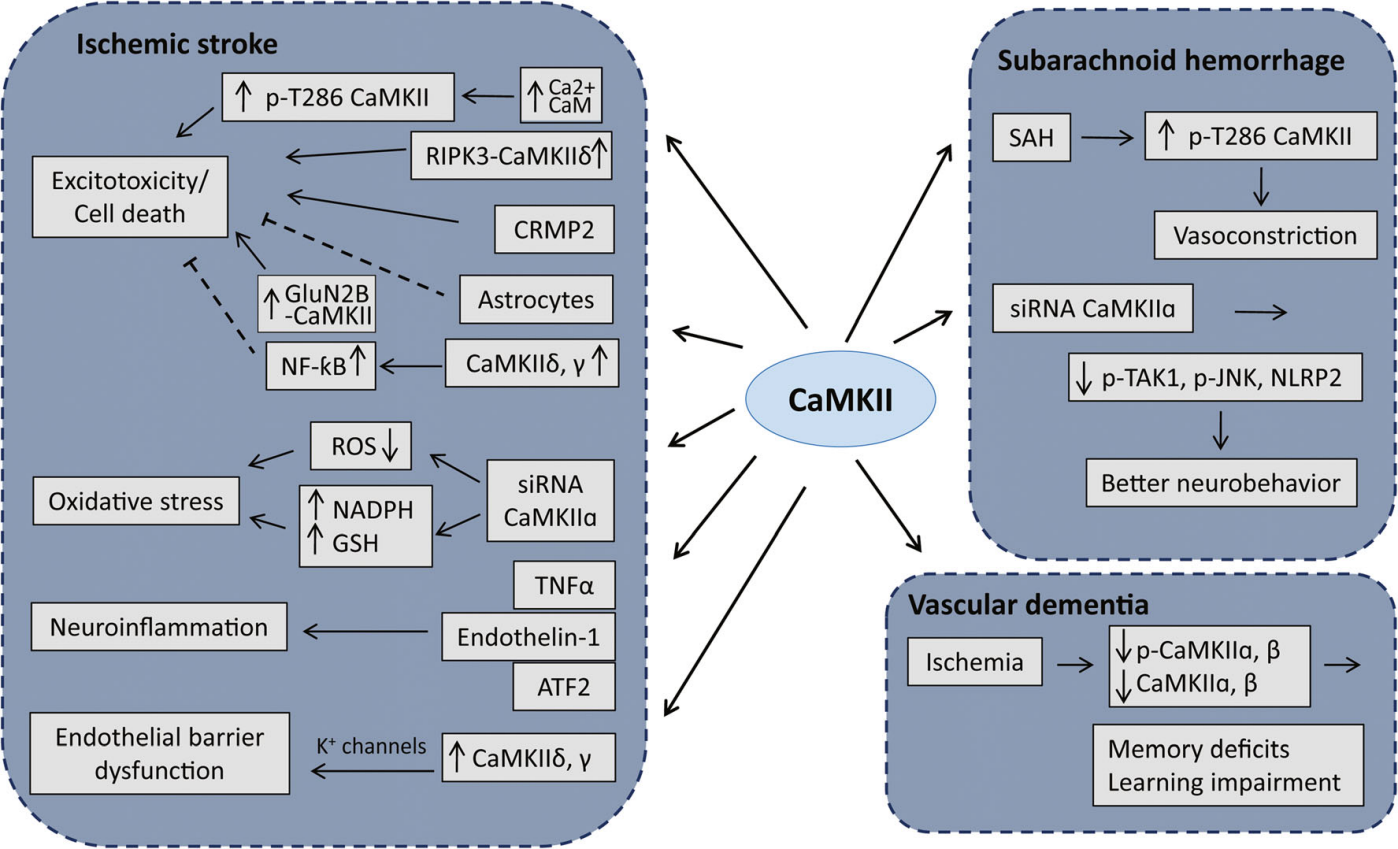
Schematic representation of key roles of CaMKII in the pathogenesis of cerebrovascular diseases. Aberrant activation or expression of CaMKII contributes to the pathophysiology of ischemic stroke by mediating the core signaling events involved in glutamate excitotoxicity, inflammation, oxidative stress, and endothelial barrier dysfunction. CaMKII is also a major regulator of vessel tone and neuronal plasticity in learning and memory and contributes to pathogenesis of subarachnoid hemorrhage (SAH) and vascular dementia

Cerebrovascular disease refers to a group of conditions or disorders that affect the blood vessels in the brain and the cerebral circulation. It is the major cause of long-term disability and stroke, the fifth leading cause of death for Americans [49]. Current studies on CaMKII in this area are mainly focused on three major cerebrovascular diseases, namely cerebral ischemia, SAH, and vascular dementia (VaD). Although we did not find any reports on CaMKII per se in other cerebrovascular diseases, such as intracranial stenosis, aneurysms, vascular malformations, etc., these diseases are common causes of ischemic or hemorrhagic stroke, for example, intracranial stenosis is the narrowing of an artery in the brain, which often leads to ischemic stroke. Similarly, aneurysms and vascular malformations create weak spots on the vessel wall, which can rupture and cause hemorrhagic stroke. In the following sections, we will discuss in details the activity, expression, signaling mechanisms, and functions of CaMKII in the three major cerebrovascular diseases (see Fig. 2 for the key roles and targets of CaMKII in these diseases).

### CaMKII and Cerebral Ischemia

Ischemic stroke accounts for 87% of all stroke cases in the USA [1]. It results in severe reduction of cerebral blood flow (CBF), lack of oxygen and nutrients in affected brain tissues, induction of excitotoxicity, and the following oxidative damage and post-ischemic inflammation, which ultimately leads to neuronal cell death and brain infarction. Untimely post-stroke reperfusion leads to secondary neuronal injury and harmful effects such as hemorrhagic transformation and blood-brain barrier (BBB) dysfunction in most stroke patients [50, 51]. Basic and translational scientific efforts have therefore been focused on the development and improvement of diagnostic and therapeutic strategies to limit the burden of ischemic insult–induced neuronal and vascular damages, such as neuronal death, BBB dysfunction, cerebrovascular endothelial injury, and vasogenic brain edema [52, 53]. As previously summarized, all four isoforms of CaMKII are expressed in the brain and regulate a wide range of neuronal and vascular functions [8]. In cerebral ischemia, altered expression and/or activation of CaMKII has been considered as a critical contributor of ischemia-induced neuronal cell death, and CaMKII activation can be targeted for both pro- or anti-neuroprotective therapies depending on timing and duration of treatments [7, 8].

#### Altered CaMKII Activity and Expression After Ischemic Stroke

##### Glutamate Excitotoxicity–Induced CaMKII Activation and Translocation

Under ischemic stroke conditions, a dramatic drop in oxygen, glucose, and ATP supply leads to uncontrolled neuronal plasma membrane depolarization, release of K^+^ into the extracellular space, and a rapid influx of Na^+^ and Ca^2+^ into the cells [54]. Neurotransmitters such as glutamate released from depolarized presynaptic and post-synaptic membranes then activate the excitatory NMDA and α-amino-3-hydroxy-5-methyl-4-isoxazolepropionic acid (AMPA) receptors [54]. Over-activated NMDA receptors in turn lead to more membrane depolarization and Ca^2+^ overloading [55]. These alterations will eventually initiate neuronal cell death via programmed cell death (apoptosis) or necrotic pathways and are given the term “glutamate excitotoxicity” [56]. The influx of Ca^2+^ through NMDA receptors after stroke rapidly activates one of the major Ca^2+^ sensors in neurons, CaMKII, via binding of Ca^2+^/CaM to its regulatory domain and leads to T286 autophosphorylation which converts it to an autonomously active and Ca^2+^/CaM-independent enzyme [57–59]. CaMKII activation and subsequent autophosphorylation can be either a neuronal damaging or pro-survival force in response to ischemic/excitotoxic insults depending on the stroke models and the timing of CaMKII activation which will be discussed in details in the following sections [7, 60].

I/R injury leads to rapid activation and translocation of CaMKII in neuronal tissues. Bing-Ren et al. have shown that isolated neocortex protein from rats after global cerebral ischemia (GCI, 15 min ischemia, followed by 30 min, 4 h, and 24 h reperfusion) exhibited increased CaMKII immunoreactivity and CaM binding that peaked at 30 min after reperfusion in the crude synaptosomal fraction of hippocampal CA1 and CA3/DG (dentate gyrus) regions but decreased in the microsomal and cytosolic fractions, suggesting a translocation of CaMKII to synaptosomes [61]. Interestingly, the translocation of CaMKII in the hippocampal CA1 region is sustained for at least 24 h after reperfusion but partially recovered in CA3/DG [61]. It has been reported by the same group that cerebral ischemia-induced CaMKII translocation to synaptic membranes may enhance neuronal firing rates and Ca^2+^ influx [62]. Similar observations were reported by Shohei et al. in a rat model of 2 h middle cerebral artery occlusion (MCAO) followed by 2 h reperfusion [63, 64]. In particular, CaMKIIα expression in crude synaptosomal fraction increased in the ischemic core area and the surrounding penumbra during ischemia and reperfusion, whereas CaMKIIα levels in cytosolic fraction decreased by 20–40% in the penumbra and by 80% in the ischemic core area [64]. Meanwhile, CaMKIIα levels remain unchanged in the whole tissue homogenates from different brain regions 2 h after reperfusion [64]. Mechanistically, the active conformation (with autophosphorylation of T286) of CaMKII can be sustained after the transient activation by binding to the NMDA receptor subunit GluN2B (also known as NR2B) [40, 60]. This interaction also leads to translocation of CaMKII from the cytosol to the membrane of PSD upon activation [35, 40, 65–67]. Additionally, it has been reported that CaMKII forms extra-synaptic clustering, a self-aggregating process that leads to reduction of CaMKII activity, under ischemic conditions and when stimulated by pathological glutamate stimuli [66–68]. I/R also results in increased phosphorylation of CaMKII on T253 in a PSD-enriched fraction prepared from rat hippocampus [18]. Although T253 does not activate CaMKII directly, it may indirectly regulate CaMKII activity by binding to other proteins, which may contribute to the persistent activation of CaMKII involved in ischemia-/excitotoxicity-induced neuronal cell death of a human neuroblastoma cell line (SH-SY5Y) [19]. In general, upon ischemic activation, CaMKII is recruited to synaptic membranes where it may modulate cellular functions that affect cell survival and neurotransmission.

##### Oxidative Stress–Induced CaMKII Activation

In addition to excitotoxicity, cerebral ischemia increases the production of ROS, such as nitric oxide and superoxide, and reperfusion further stimulates ROS generation from neurons and glia cells, resulting in CaMKII oxidation and activation [69]. Erickson et al. reported that M281/282 oxidation leads to a similar conformational change in CaMKII produced by T286 phosphorylation that could maintain CaMKII in an open position to phosphorylate its substrates after Ca^2+^/CaM dissociation [70]. In another study, extracts from mouse forebrain synaptosomes were treated with different oxidizers and immunoblotted under non-reducing conditions with an antibody against CaMKIIα showing extensive oxidation of CaMKII forms large molecular weight aggregates and oxidized CaMKII is more closely associated with PSD [71]. To better investigate ischemia-induced oxidation of CaMKII under physiological conditions, the authors soaked acutely prepared mouse hippocampal slices in a modified artificial cerebrospinal fluid buffer pre-saturated with 95% N_2_/5% CO_2_ to mimic the ischemia condition, and a similar oxidative modification of CaMKII was observed in the hippocampal slices exposed to ischemic insults [71]. It was also noted in this study that oxidation of CaMKII suppressed both total (in the presence of Ca^2+^/CaM) and autonomous (independent of Ca^2+^/CaM) kinase activities and synaptic potentiation post ischemia due to the formation of large aggregates [71]. CaMKII oxidation has been extensively studied in cardiovascular and pulmonary diseases where oxidative stress drives disease pathology (see a detailed review by Mark E. Anderson [37]). Contrary to the rapid activation of CaMKII, decreased CaMKII activities were also documented in cultured rat hippocampal neurons treated with glutamate [58] and in the rat neocortex, striatum, and hippocampus after forebrain ischemia [72]. The later study also reported that protein levels of CaMKIIα and β decreased in the cytosol and increased in the particulate fractions of the three brain regions they examined, suggesting an intracellular redistribution of CaMKIIs [72]. Nonetheless, functional outcomes or pathological consequences of excitotoxic/ischemia-induced loss of CaMKII activity were not discussed in these two studies.

##### Altered CaMKII Expression at Transcriptional Level

Besides post-translational regulation by phosphorylation or oxidation, we recently showed that CaMKII could also be regulated at transcriptional level by two lncRNAs, *C2dat1* and *C2dat2*. Wild-type C57BL/6J mice were subjected to 1 h of MCAO and 6–24 h of reperfusion. *C2dat1* expression was significantly increased in the cortical penumbra at 6–24 h post reperfusion compared to the sham-operated mice. This was also accompanied by an increased expression of CaMKIIδ at transcript and protein levels [47]. Similar expression patterns can be observed in primary mouse neuron cultures under *in vitro* ischemic condition [47]. Moreover, knockdown of *C2dat1* and *2* by siRNAs significantly blocked *in vitro* ischemia-induced expression of *CaMK2D* in N2a cells and primary neurons. The knockdowns also exacerbated ischemia-induced neuronal cell death [47, 48]. However, knockdown of *C2dat1* and *C2dat2* had no effects on oxygen-glucose deprivation and reoxygenation (OGD/R)–induced expression of CaMKIIγ, indicating specific targeting of *CAMK2D* by these lncRNAs [48]. The upregulation of CaMKIIγ was also interesting as both CaMKIIδ and γ were thought to be absent or low in neurons. Knockdown of CaMKIIδ and γ exacerbated OGD/R-induced neuronal cell death, implying that both isoforms promote neuronal survival. This is distinct from its pro-death effects observed in the early phase of I/R. It is possible that increased CaMKIIδ and γ may alter the functionality of CaMKII holoenzyme since their upregulation could change several properties of CaMKII including the ratio of subunit composition in the holoenzyme, stimulated/autonomous activity, localization, and protein interactions.

#### Contributory Roles of CaMKII in the Pathogenesis of Ischemic Stroke

Cerebral ischemia triggers a cascade of pathological events that ultimately result in irreversible neuronal injury in stroke-affected brain tissues within minutes of stroke insult [54]. The pathophysiological order of the events ranges from excitotoxicity within minutes, robust inflammatory responses within hours, to programmed cell death and tissue loss within hours and days of stroke onset [54, 73, 74]. Based on our literature search, it appears that most studies have been focusing on the involvement of CaMKII in excitotoxicity-induced neuronal death [7, 60, 75, 76]. Only a few reported functional roles of CaMKII in inflammation [77], endothelial barrier integrity [3], and astrocyte dysfunction [78] after experimental stroke (summarized in Table 1). In this section, we review the functional inputs of CaMKII in these biological processes.

**Table 1:** Contributory roles of CaMKII in ischemic stroke: a summary of pathological events, their cellular targets, and the overall pathological outcome of CaMKII

	Pathological events in stroke	Targeting sites	Cellular targets/outcomes
CaMKII	Increased pCaMKII/CaMKII ratio after OGD/R	Neurons	Pro-apoptosis [79]
CaMKII	Prolonged CaMKII activity inhibition exacerbates excitotoxicity	Neurons	Pro-apoptosis [75]
CaMKIIɑ	siRNA targeting CaMKIIɑ reduces oxidative stress after MCAO	Neurons Astrocytes	Glucose 6-phosphate dehydrogenase [80]/pro-death
CaMKII	CaMKII inhibitor KN93 reduces inflammatory marker in organ culture	Cerebral arteries	TNF-α receptor 1 (77)/pro-death (implied)
CaMKIIδ	OGD induces increased RIPK3-CaMKIIδ interaction	Oligodendrocyte progenitor cells	Pro-necroptosis [81]
CaMKIIɑ	Increased p-T286 CaMKIIɑ in cerebellar vermis after GCI	Cerebellar vermis	Decrease Purkinje cell density [82]/pro-death
CaMKIIδ, γ	Increased potassium currents, results in hypoxia-induced cell swelling of brain endothelial cells	ECs	Voltage-gated K^+^ channels [3]/pro-death
CaMKIIɑ	CaMKIIɑ^−/−^ rat presents larger infarction after 2.5 h MCAO and 21 h reperfusion	Whole body	Neuroprotection [83]
CaMKII	CaMKII activity inhibition at the time of glutamate insult can be neuroprotective	Neurons	Neuroprotection [60]
CaMKII	CaMKII inhibition by tat-CN21 induces calcium oscillations in cortical astrocytes	Astrocytes	Neuroprotection [84]
CaMKIIδ, γ	I/R*-*induced CaMKIIδ, γ promotes neuronal survival	Neurons	NF-κB [48]/neuroprotection

##### Excitotoxicity-Induced Cell Death

Cerebral ischemia–induced neuronal damage is largely caused by glutamate-mediated excitotoxicity. Synaptic glutamate released from the damaged neurons leads to the activation of NMDA and AMPA receptors [85, 86]. NMDA receptors are Ca^2+^ permeable, and the opening of these channels leads to further membrane depolarization and greater Ca^2+^ influx which exacerbates intracellular Ca^2+^ overload [86]. Oxygen deprivation–induced acidosis disrupts Na^+^/Ca^2+^ exchanger, prevents the efflux of Ca^2+^, and further contributes to Ca^2+^ overload. It is known that Ca^2+^, at a lower concentration (~ 10^−6^ M), mediates neuroprotective processes, whereas at higher concentration about 10^−4^–10^−3^ M, Ca^2+^ induces apoptosis or necrosis [87, 88]. Prolonged Ca^2+^ increases during ischemia result in pathophysiological activation of many intracellular signaling cascades, including Ca^2+^/CaM/CaMKII [88]. CaMKII also phosphorylates NMDA and AMPA glutamate receptors, which further increases Ca^2+^ influx and aggravates excitotoxicity [88]. These processes contribute to neuronal death during I/R but may also contribute to the death of endothelial cells (ECs) [74], astrocytes [89, 90], and oligodendrocytes [81] after cerebral I/R insult or OGD/R. Excitotoxicity may induce cell death mainly by necrotic, apoptotic, or autophagic pathways.

##### Necrosis

Necrosis is generally identified as the cells present swollen organelle phenotypes, which subsequently burst to release their contents into the extracellular space, whereas apoptotic cells undergo nuclear and cytoplasmic condensation, followed by cell fragmentation and phagocytosis by phagocytes [91]. Necrosis occurs during first post-stroke minutes, primarily in the ischemic core [92, 93], whereas apoptosis develops later, for hours or days, mainly in the surrounding penumbra. Overloaded cytosolic Ca^2+^ can induce necrosis via activation of proteases, phospholipases, and mitochondrial permeability transition [94]. The best-characterized form of regulated necrosis is necroptosis. Necroptosis is defined as a necrotic cell death dependent on the kinase activity of Receptor Interacting Protein 1 (RIP1) and RIP3 and expression of mixed lineage kinase–like protein [94, 95]. Increased RIPK3 expression and RIPK3-CaMKIIδ interaction have been reported in a study in which necroptosis is induced *in vitro* in oligodendrocyte progenitor cells (OPCs) by OGD plus caspase inhibitor zVAD treatment to facilitate cell death from apoptosis to necroptosis [81], whereas RIPK3 siRNA treatment significantly reduced RIPK3 expression, the RIPK3-CaMKIIδ interaction, and OPC cell death at 24 h after OGD [81]. Moreover, activated CaMKIIδ (p-T287) levels were increased in OPCs at 12 and 24 h after OGD, whereas total CaMKIIδ levels were not altered. Furthermore, RIPK3 inhibition via siRNA decreased p-T287 CaMKIIδ levels [81]. These results suggest that RIPK3 mediates oligodendrocyte necroptosis through interacting with and activating CaMKIIδ. In another study by Lixuan et al., they found in a rat model of transient global cerebral ischemia, pretreatment of KN93 (a CaMK inhibitor) before the surgery significantly reduced RIP1 and RIP3 interaction and protected hippocampal neurons from global cerebral ischemia–induced necroptosis [96].

##### Apoptosis

Apoptosis plays a major role in tissue loss in ischemic lesions. Unlike in the ischemic core area of the stroke where neuronal death is generally mediated by necrosis and is considered beyond rescue, apoptosis is the dominating cell death modality in the penumbra [97]. The therapeutic time window between the onset of stroke insult and apoptosis-induced tissue loss surrounding the ischemic site makes it a very attractive target for stroke therapy [97–99]. Diverse apoptotic initiation and regulation pathways are induced in penumbra from different initial triggers and various anti-apoptotic proteins are upregulated as well. CaMKII is found to be upregulated in the penumbra surrounding the infarction core in the rat cerebral cortex at 1, 4, and 24 h after stroke [99]. Our group has also discovered increased CaMKIIδ fluorescence intensity in the peri-infarct region after I/R [47]. Nicholas et al. reported an increase in CaMKIIɑ activity (p-T286) in mouse cerebellar vermis at 6 h after global I/R condition. This is believed to contribute to decreased Purkinje cell (a type of neuron located in the cerebellum) densities at 7 days after surgery because it can be inhibited with a single dose of a peptide inhibitor of CaMKII activity, tat-CN19, at 30 min after surgery [82]. In another *in vitro* study in which PC12 cells were incubated under OGD condition for 2 h and then cultured under normal conditions for 1, 2, 4, 5, 12, and 24 h, pCaMKII/CaMKII ratio increased from 1 h after incubation in normal conditions, reached maximum at 2 h, and then gradually declined with culturing time but remained higher than non-OGD control group [79]. Cell apoptosis increased in the OGD/R-induced PC12 cells and was blocked by KN93 treatment [79]. These studies suggest that CaMKII activation following I/R contributes to apoptotic cell death. This is consistent with previous studies that have identified a pro-apoptotic role for CaMKII activation following ischemia [18, 19, 100–102] (also summarized by Steven et al. [7]).

Despite the overwhelming evidence supporting a pro-apoptotic function of CaMKII after I/R, some studies suggest that CaMKII can be neuroprotective by phosphorylating and inhibiting several pro-apoptotic proteins such as NO synthase, Bad, and GSK-3 [7]. In these studies, CaMKII was mostly studied as a whole without distinguishing the specific isoforms involved. Therefore, the role that each CaMKII isoform played in neuronal survival after ischemia remains to be defined. In our studies, both CaMKIIδ and CaMKIIγ were upregulated in the penumbral tissue from 1 to 4 days after MCAO surgery [47, 48]. Overexpression of CaMKIIδ promoted neuronal survival in the OGD/R model, while knockdown of CaMKIIγ resulted in significant neuronal death after OGD/R [48]. We also demonstrated that I/R-induced CaMKIIδ and CaMKIIγ promoted neuronal survival through activating the canonical NF-κB signaling pathway [48]. It is notable that the induction of CaMKIIδ by lncRNAs was gradual and persistent which is distinct from the acute activation of CaMKII immediately after I/R that often associates with neuronal death, highlighting the dynamic regulation and functional diversity of the CaMKII kinases in the course of I/R injury in the brain. In line with the pro-survival function of CaMKII, there are also studies that suggest that prolonged inhibition or knockout of CaMKII exacerbates ischemic injuries [60, 75, 83]. For example, genetic knockout of CaMKIIɑ resulted in larger infarction compared to wild-type littermates in a MCAO model [83]. This finding could be explained by the developmental defects caused by lacking CaMKIIɑ since it was reported by another group that CaMKIIɑ knockout mice are epileptic [103], meaning the KO mice are generally more susceptible to ischemic insults. The Hudmon group showed that prolonged pharmacological inhibition of CaMKII (incubate with tat-CN21 for 4–24 h) exacerbated excitotoxicity following glutamate/glycine insult in cultured neurons [60]. They further confirmed that cultured neurons underwent apoptosis in response to prolonged CaMKII inhibition (>4 h) since the prolonged CaMKII inhibition was associated with an increase in TUNEL staining and caspase-3 cleavage [75]. These findings are consistent with the notion that the extent of neuronal damage in the penumbra depends on the level of CaMKII, and loss of CaMKII augments neuronal susceptibility to ischemic insults.

##### Autophagy

Excitotoxic insults also lead to neuronal autophagy, and accumulating studies have indicated autophagy is involved in the pathophysiological changes in ischemic stroke [104, 105]. Interestingly, autophagy can play both protective and detrimental roles in ischemic stroke which are summarized in a review article by Pei et al. [106]. CaMKII has been shown to regulate autophagy in neuroblastoma cells. Li et al. reported that CaMKII can directly phosphorylate Beclin-1 (a critical regulator of autophagy) to promote the activation of autophagy [107]. CaMKII has also been shown to regulate autophagy in myocardial I/R injury. Kong et al. demonstrated that CaMKII inhibition attenuated autophagic flux through mitigating the phosphorylation of Beclin-1 [108]. In addition, Kulbe et al. found that tat-CN21, a highly selective peptide inhibitor of CaMKII with neuroprotective function [109], inhibited basal autophagy in primary hippocampal neurons [110]. However, in the presence of excitotoxic glutamate insults that blocked autophagy, no further effects were observed after tat-CN21 treatment [110]. Taken together, although a promising target, more investigations are needed to define the role of CaMKII and its regulatory mechanisms in autophagy in cerebral I/R injury.

##### Neuroinflammation

Cerebral ischemia induces a cascade of molecular events that transform the cerebrovascular endothelium from a quiescent to a proinflammatory state [111]. Activated cerebral endothelium have the capacity to produce and/or secrete many inflammatory mediators, most important of which are the selectins, proinflammatory cytokines, and integrins, thus becoming a source of inflammation themselves in the cerebral vasculature [112]. Tumor necrosis factor alpha (TNF-α) is one of the proinflammatory cytokines that initiates inflammation in many cell types via its receptor (TNF-α receptor 1) and plays an essential role in cerebrovascular inflammation. Using the method of organ culture for inducing ischemic-like vascular wall changes, Roya et al. incubated rat basilar arteries in serum-free medium for 0, 3, 6, or 24 h in the presence or absence of CaMKII inhibitor KN93 [77]. They found CaMKII activation in the non-incubated (0 h) arteries was gradually decreased with time. The TNF-α receptor 1 expression was elevated after organ culture and can be blocked by treatment of CaMKII inhibitor KN93 [77]. Their findings suggest that inhibiting CaMKII activation attenuates neuroinflammatory mediators and could contribute to neuroprotection. The elevation of another proinflammatory mediator, endothelin-1 (ET-1), has been indicated in the regions of vascular injuries and inflammation [113]. In mouse brain microvascular endothelial cells bEnd.3, Chih-Chung et al. found that ET-1 stimulated increases of intracellular Ca^2+^. This contributed to ET-1–induced CaMKII activation and ET-1/ET receptor–mediated cyclooxygenase-2/prostaglandin E2 release (both are known to aggravate brain inflammation) through regulating mitogen-activated protein kinases and the downstream activating transcription factor 2 [114]. Both studies suggest that strategies targeting CaMKII may be beneficial for brain injury and inflammatory disease.

##### Oxidative Stress–Induced Tissue Damage

Many pathological events occur during the ischemia reperfusion procedure, including the release of free radicals that bring damages to the brain and aggravate neuronal injuries. These free radicals represented by ROS could spark oxidative stress in the brain. With the help of glutathione reductase and nicotinamide adenine dinucleotide phosphate (NADPH), oxidized glutathione can be transformed to reduced glutathione, which is necessary to eliminate excess ROS [115]. Previous studies indicate that CaMKII can be activated by ROS, and activated CaMKII also regulates redox regulators such as NADPH and reduced glutathione to further exacerbate oxidative stress–induced tissue damage [37], providing a potential feed-forward cycle for CaMKII to infuse the oxidative stress–induced tissue damage. In a rat ischemic stroke model, Yamin et al. performed intracerebroventricular injection of siRNA that targets CaMKIIα at 48 h pre-MCAO surgery. They found rats that received siRNA injection showed milder neurological deficits and smaller infarct volume compared to those that received scramble control injection [80]. In addition, CaMKIIα siRNA decreased ROS content and increased the reduced glutathione/oxidized glutathione and NADPH/NADP^+^ ratios in the rat cortex after MCAO [80], suggesting that CaMKIIα inhibition could suppress oxidative stress through upregulating NADPH and reduced glutathione. This study provides evidence that CaMKII inhibition can serve as anti-oxidant therapy in cerebrovascular disease.

##### Endothelial Barrier Dysfunction

Endothelium plays a critical role in the regulation of vascular function. Untimely reperfusion after ischemic stroke often times leads to harmful consequences including blood-brain barrier (BBB) dysfunction. BBB is centrally positioned within the neurovascular unit, and several elements contribute to the physical barrier of BBB, including cerebrovascular ECs, junctional proteins, pericytes, astrocytes, and basement membrane [53]. Among those, cerebrovascular ECs are the primary barrier between circulation and tissue that play an essential role in the maintenance of BBB integrity/permeability and cerebral homeostasis. CaMKIIα was found in the perinuclear region of the cells in primary cerebral EC culture [116]. In a separate study, glutamate treatment induced CaMKIIα activation in primary cultures of rat cerebral ECs even after 10- and 60-min recovery [59]. Besides CaMKIIα, both CaMKIIδ and γ are expressed in rat brain capillary ECs. Moreover, activation of CaMKIIδ and γ was found upstream of voltage-gated potassium channels, resulting in hypoxia-induced cell swelling that may precede barrier dysfunction [3]. In addition, CaMKIIδ and γ were found in human central nervous system pericytes, and their activation is required for extracellular acidosis–induced cAMP-response element binding protein activation and proinflammatory cytokine interleukin-6 upregulation [4]. CaMKII is also found expressed in cultured cortical astrocytes using pan-CaMKII antibody [84]. To mimic the rapid loss of CaMKII activity in brain tissue during an ischemic insult, cultured cortical astrocytes were treated with CaMKII inhibitor tat-CN21. Inhibiting CaMKII activity in neuronal support cells (astrocytes) induces ATP release from astrocytes and contributes to Ca^2+^ oscillations in astrocytes and induces neuronal death [84]. However, functional roles of CaMKII in regulating endothelial barrier function *in vivo* remain to be explored.

#### Inhibitors of CaMKII and Their Effects on Post-insult Neuroprotection

Given the well-documented CaMKII activation immediately after ischemic insults and its role in mediating neuronal death in experimental models of stroke, it appears to be a promising target for acute clinical intervention to limit brain damage after stroke. Small molecule CaMKII inhibitors KN62 and KN93 have been widely used to study CaMKII function *in vitro*, as they are membrane penetrating [7]. However, KN inhibitors are not selective inhibitors for CaMKII activation [117]. A more selective CaMKII inhibitor is CN21, which is a 21mer peptide derived from the natural CaMKII inhibitory protein CaM-KIIN [118]. Peptide-based inhibitors are further made cell penetrating by fusion with a sequence motif such as tat [119]. Inhibiting stimulated and autonomous CaMKII activity with tat-CN21 attenuated neuronal cell death after a glutamate insult *in vitro* or *in vivo* and was effective even when administered 1 h after the MCAO surgery, which is within a clinically relevant window of therapeutic opportunity [109]. In a separate study in which KN93, tat-AIP, and tat-CN21 were applied either immediately before or after the excitotoxic insult, post-insult neuroprotection by CaMKII inhibition was confirmed using both tat-CN21 and tat-AIP, but not KN93 [60]. This study also confirmed significant neuroprotection in cortical cultures when tat-CN21 was administered 2 h after excitotoxic challenge. On the contrary, KN93 was neuroprotective only when administered during but not after the insult in both studies [60, 109]. In a rat GCI model, tat-CN21 administered 3 h after reperfusion exerted neuroprotective effects against delayed hippocampal CA1 pyramidal neuronal cell death 10 days post GCI through decreasing GCI-induced phosphorylation, translocation, and membrane targeting of CaMKIIɑ, as well as CaMKIIɑ-NR2B interaction [102]. Mechanistically, tat-CN21 and tat-AIP can both block Ca^2+^/CaM-stimulated activity of CaMKII and the Ca^2+^-independent autonomous CaMKII activity during the insult, whereas KN93 only inhibits Ca^2+^/CaM–stimulated CaMKII activity effectively, suggesting that the autonomous activation of CaMKII may be a promising drug target for post-stroke neuroprotection. However, both KN93 and AIP have been shown to have off-target effectors. KN93 is an inhibitor of several CaMK family members including CaMKI, CaMKII, CaMKIV, and voltage-gated K^+^ and Ca^2+^ channels [60]. The peptide inhibitor AIP has also been shown to inhibit other CaMK family members [60].

Interestingly, using a competitive inhibitor of CaMKII kinase activity to inhibit ischemia-induced sustained activation of CaMKII may lead to opposite effects. For example, tat-CN21 treatment for 8 h or more before the glutamate insult resulted in a significant increase of neuronal death in cortical cultures compared to glutamate insult alone [60]. In an *in vitro* study, tat-CN21 treatment in cortical astrocytes decreased glutamate uptake, increased ATP release, and dysregulated astrocyte-neuron signaling thereby compromising neuronal survival after ischemic insult [84]. These studies are again in line with the previous observation that CaMKIIɑ^−/−^ mice had larger infarction than the wild-type mice after MCAO [83]. Overall, CaMKII inhibition–mediated neuroprotection was achieved when inhibitors were administered at the acute phase of ischemic/excitotoxic insults, whereas sustained CaMKII inhibition long (> 4 h) before ischemic insult could exacerbate neuronal death by enhancing neuronal vulnerability to the insults.

### CaMKII and Subarachnoid Hemorrhage

Hemorrhagic stroke is caused by rupture of weakened arterial blood vessels, causing bleeding and damage to the surrounding brain tissue. There are two types of hemorrhagic stroke; the most common type is intracerebral hemorrhage (ICH) that occurs within the brain tissue, and the less common type is subarachnoid hemorrhage (SAH). There have not been any studies on CaMKII in ICH. Therefore, we mainly discuss the role of CaMKII in SAH in this section. Approximately 80% of non-traumatic, spontaneous SAH is due to the rupture of an intracranial aneurysm leading to the extravasation of arterial blood into subarachnoid space [120]. The initial mortality in SAH patients before receiving medical attention is high (12%) [121]. After several days, the development of delayed cerebral ischemia and cerebral vasospasm contributes to a prolonged CBF reduction in approximately 40% of SAH patients [122]. Of those patients who survive SAH, many of them suffer from long-term memory and cognitive defects that severely impact their quality of life [123].

Early brain injury (first 72 h after SAH) could happen within minutes after the initial bleeding, mostly due to ischemia, based on a large body of animal studies [124, 125]. A study by Parker et al. utilized quantitative mass spectrometry–based phosphoproteomic analysis to dissect early SAH-induced phosphorylation events (0.5 h and 1 h post SAH) in cerebral artery tissue lysates from SAH rats [126]. Among those protein kinases significantly altered by SAH, they found CaMKII activation at its autophosphorylation site T286 which could be blocked by treating the rat with KN93 prior to SAH. However, the total CaMKII expression remained unchanged [126], implying that CaMKII might play a role in SAH-induced cerebral vasculopathy. In agreement with Benjamin’s study, Lars et al. also observed a rapid activation of CaMKII at its autophosphorylation site T286 at 1 h post SAH in rat cerebral artery lysates, and the activated CaMKII reduced to sham-operated levels at 6 and 24 h post SAH [127]. They also observed an increased overall CaMKII protein expression at 72 h post SAH by immunohistochemistry in cerebral vascular smooth muscle cells. Interestingly, this can be blocked by KN93 treatment prior and immediately after SAH operation [127]. Moreover, KN93 treatment attenuated endothelin and serotonin receptor–mediated vasoconstriction and improved sensorimotor function at 48 h post SAH. Therefore, inhibiting CaMKII activation by KN93 can be beneficial through reducing cerebral vasoconstriction and improving neurological outcomes after SAH [127]. The beneficial effects through inhibiting CaMKII activity were further affirmed by another group using siRNA that targets CaMKIIα [128]. The expression of activated CaMKIIα in rat brain increased from 3 to 24 h post SAH (total CaMKIIα expression did not change), and p-CaMKIIα (T286) mainly localized within the neurons and microglia [128]. Meanwhile, CaMKIIα knockdown by siRNA injected intracerebroventricularly at 48 h prior to surgery, markedly improved neurobehavior after SAH [128] and significantly decreased p-TAK1, p-JNK, and nucleotide-binding domain-like receptor protein 3 (NLRP3) inflammasome expression (indicating reduced inflammatory response). Sang et al. reported that 6 days after SAH injury, CaMKII overall expression was reduced in the dendritic layer of the CA1 area of the hippo-pyramidal cells. This was accompanied by a significant decrease of synapses in neurons in the CA1 area that could be responsible for loss of long-term potentiation (a persistent strengthening of synapses following high levels of neuronal stimulation) after SAH [129]. Taken together, SAH-induced CaMKII activation or overall expression can vary depending on different brain regions, cell types, SAH models, and time after SAH surgery. Nonetheless, inhibition of CaMKII activity mostly associates with improved neurological outcome after SAH.

### CaMKII and Vascular Dementia

Vascular dementia (VaD) is a complex neurodegenerative disorder that is manifested as the functional consequence of reduced blood flow (acutely or chronically) in the brain [130]. VaD associates with a progressive cognitive and memory impairment, and the instances increase with age [131]. While CaMKII is highly enriched in neuronal tissue (especially in hippocampal pyramidal cells), the autophosphorylation of CaMKII is deemed essential for the hippocampus-dependent memory formation and the long-lasting increase in synaptic efficacy following long-term potentiation [132]. The activation and functional involvement of CaMKII signaling in the hippocampus and other brain regions has been well documented in different animal VaD models.

Most studies focus on the neuroprotective function of CaMKII in hippocampal CA1 pyramidal neurons and the restored learning and memory ability in VaD animals through activating CaMKII. For example, in a VaD mouse model, Yui et al. reported a cerebral ischemia–induced contextual memory deficit, and both activated CaMKII (including α and β isoforms) and the total CaMKIIα and CaMKIIβ expressions were significantly decreased in the hippocampal CA1 region at 12 days in bilateral common carotid artery occlusion (BCCAO) mice [133]. Under certain BCCAO conditions, they found nobiletin (a compound for Alzheimer’s disease therapy) treatment inhibited delayed neuronal death and restored both CaMKII (α and β) activation and the overall expression in the hippocampal CA1 region. Nobiletin also improved BCCAO-induced memory deficits and hippocampal long-term potentiation impairment [133]. Xiao-Juan et al. reported similar observations that cerebral ischemia led to reduced p-CaMKII (T286) in the hippocampal CA1 pyramidal neurons, which associates with decreased spatial learning and memory capacity of mice subjected to right unilateral common carotid artery occlusion (a chronic VaD model) at 30 days after surgery [134]. These deficits can be partially rescued by CaM antagonist loaded micelle treatment [134]. The same group reported in another study using a bilateral common carotid artery stenosis mouse model (BCCAS, a common model of cerebral hypoperfusion–induced cognitive impairment) that CaM inhibitor DY-9836 restored the decrease of p-CaMKII (T286) in hippocampal pyramidal neurons and improved learning impairment after BCCAS surgical operation [135]. Similar results were reported in a rat permanent BCCAO model [136, 137]. In addition, VaD is an age-related disease. Qi et al. explored aging-induced cognitive deficits in a senescence-accelerated mouse (SAMP8) model [138], in which the cognitive deficits can be observed as early as 4 months after birth [139]. A significant decrease in p-CaMKIIα (T286) but not the non-phosphorylated CaMKIIα expression, was observed in the prefrontal cortex region of older SAMP8 mice compared to the younger SAMR1 mice [138], and oral administration of a traditional Chinese prescription, Kangen-karyu, ameliorated aging-induced memory deficits and restored p-CaMKIIα expression in the cerebral cortex of older SAMP8 mice [138]. Despite the variety of VaD models employed in different studies, it appears that they all come to the same conclusion that restoring p-CaMKII (T286) or CaMKII overall expression is beneficial for cerebral ischemia–/age-induced cognitive deficits.

## Summary and Future Prospects

In this article, we reviewed the important roles of CaMKII serine/threonine kinases in cerebrovascular diseases. There are both well-studied and under-studied areas of CaMKII functions in stroke, subarachnoid hemorrhage, and vascular dementia (summarized in Table 2). The four isoforms of CaMKII, although highly conserved in domain structures, vary widely in expression patterns in different tissues and exhibit significant pathobiological functional diversity in cerebrovascular diseases. In light of all the literature on this topic, we have come to the conclusion that CaMKIIs significantly influence the pathological processes of cerebrovascular diseases by regulating a whole host of events and neurovascular cells including neurons, ECs, smooth muscle cells, pericytes, and astrocytes. In addition to the most extensively studied isoform CaMKIIɑ, which is a neuron-specific isoform, the understudied CaMKII isoforms (CaMKIIδ and γ) have also emerged as new players in neuronal recovery after ischemic insults [47, 48, 81]. Overall, in ischemic stroke, there is compelling evidence supporting rapid CaMKII activation immediately after I/R insults, and activated CaMKII plays a crucial role in mediating excitotoxicity-induced neuronal death through necrotic, apoptotic, and possibly autophagic pathways and contributes to secondary oxidative damage, neuroinflammation, and BBB dysfunction, indicating that inhibition of CaMKII may be a promising therapeutic strategy to limit brain damage in acute phase of ischemic injury. However, there remain discrepancies in the role of CaMKII in later phases of ischemic stroke with evidence showing that prolonged inhibition or loss of CaMKII compromises neuronal function and survival, suggesting a positive role of CaMKII in neuronal recovery. In SAH, despite the varied CaMKII activity and expression in different brain regions after hemorrhagic injury, inhibition of CaMKII activity has consistently resulted in improved neurological outcome after SAH, supporting a pro-death function of CaMKII in SAH. In contrast, results from various VaD models have consistently demonstrated that increased p-CaMKII (T286) or CaMKII overall expression reduces cerebral ischemia–/age-induced cognitive deficits, implying that restoration of CaMKII activity and expression may benefit VaD patients. From a therapeutic perspective, the timing and duration of CaMKII inhibition will need to be calibrated carefully since inhibitors administered at the acute phase of I/R may be neuroprotective, while those resulted in long periods of sustained CaMKII inhibition could exacerbate neuronal death. Additionally, CaMKII inhibitors that block autonomous activation of CaMKII clearly provide added advantage for post-stroke neuroprotection, such as tat-CN21 which is a selective and efficacious CaMKII inhibitor that blocks both stimulated and autonomous activity of CaMKII *in vitro* and *in vivo*.

**Table 2:** CaMKII studies in cerebral vascular diseases: a summary of well-studied and under-studied areas of CaMKII in major cerebral vascular diseases

CaMKII	Well-studied	Under-studied
Ischemic stroke	• Changes of CaMKII activity, expression, and localization before and after ischemic stroke • Ischemia-induced CaMKII oxidation • Role of CaMKII holoenzyme or CaMKIIα in ischemia-induced apoptotic neuronal cell death • CaMKII in excitotoxicity-induced neuronal death in ischemic stroke • Effects of CaMKII inhibitors on post-insult neuroprotection	• Signaling mechanisms of CaMKII in neurons and non-neuronal cells in ischemic stroke • Functions of CaMKII in non-neuronal cells such as vascular endothelial cells • Involvement of CaMKII in neuroinflammation and endothelial barrier dysfunction induced by ischemic insults • Roles of under-studied CaMKII isoforms such as δ and γ in ischemic stroke • CaMKII regulation by non-coding RNAs • Translation of CaMKII-targeted agents to clinical settings
Subarachnoid hemorrhage	• Changes of CaMKII expression and activity before and after SAH • Impact of CaMKII activation on neurological outcome after SAH	• How CaMKII contributes to improved neurological outcome after SAH • Functional involvement and signaling mechanisms of CaMKII in neurons and non-neuronal cells after SAH
Vascular dementia	• Activation and neuroprotective function of CaMKII in hippocampal neurons in different animal VaD models	• Mechanisms of CaMKII activation/upregulation in VaD • Functional involvement of CaMKII in non-neuronal cells in VaD models

Although previous studies have implied the effectiveness of neuroprotectant in animal stroke models, there has not been much progress made by far in regard to the successful translation of neuroprotective strategies to clinical settings [73], suggesting that focusing only on neuroprotection is insufficient. In other words, non-neuronal cells and the local microenvironment of the surviving neurons could serve as potential therapeutic targets to alleviate ischemic brain injuries as well. For example, cerebrovascular ECs play an essential role in the maintenance of BBB integrity and cerebral homeostasis under physiological conditions [140, 141]. Ischemia-induced endothelial injury increases cerebrovascular permeability and compromises BBB integrity. Proinflammatory factors may then pass through the compromised BBB to attract circulating immune cells to the injured brain which leads to secondary ischemic brain parenchymal injuries [142]. Studies by Ferenc group indicated that CaMKIIɑ, δ, and γ are all expressed in murine cerebrovascular ECs [59, 116], and Blasig group further reported that hypoxia induced activation of CaMKIIδ and γ in rat brain capillary ECs associated with cell swelling that may precede barrier dysfunction [3]. Based on this previous evidence, it would be of great interest to explore whether CaMKII is one of the upstream molecular regulators of BBB stability, dysfunction, or protection after cerebral ischemia. Moreover, studies in rodent cerebral ischemia models have demonstrated that cerebral vascular ECs begin to proliferate in the peri-infarct regions as early as 12h after stroke onset [143]. Importantly, the extent of angiogenesis is often closely associated with reduced cerebral infarction and improved neurological recovery [143]. Thus, studying whether CaMKII activity is involved in post-ischemic angiogenesis may be a useful therapeutic strategy for treatment of ischemic stroke in clinical settings.

With the help of advanced techniques such as genetic manipulation, genomic editing, and proteomics, more comprehensive and in-depth understanding of CaMKII’s roles in cerebrovascular diseases will be conducive to develop novel therapeutic approaches to effectively reduce the risk of cerebrovascular diseases, as well as improve prognosis and decrease complications.

## Data Availability

All data and materials are available and support the published claims and comply with field standards.

## Abbreviations

CaMKII: Ca^2+^/calmodulin–dependent protein kinase II
CaM: calmodulin
CBF: cerebral blood flow
BBB: blood-brain barrier
MCAO: middle cerebral artery occlusion
OGD: oxygen and glucose deprivation
NLS: nuclear localization sequence
PSD: post-synaptic density
ROS: reactive oxygen species
lncRNA: long non-coding RNA
GCI: global cerebral ischemia
EC: endothelial cell
SAH: subarachnoid hemorrhage
VaD: vascular dementia
BCCAO: bilateral common carotid arteries occlusion
NMDA: N-methyl-D-aspartate
AMPA: α-amino-3-hydroxy-5-methyl-4-isoxazolepropionic acid
NADPH: nicotinamide adenine dinucleotide phosphate
TNF-α: tumor necrosis factor alpha
ET-1: endothelin-1

## Code Availability

Not applicable. No coding in the study.
